# Acceleration of Wound Healing through Amorphous Calcium Carbonate, Stabilized with High-Energy Polyphosphate

**DOI:** 10.3390/pharmaceutics15020494

**Published:** 2023-02-02

**Authors:** Shunfeng Wang, Meik Neufurth, Hadrian Schepler, Rongwei Tan, Zhending She, Bilal Al-Nawas, Xiaohong Wang, Heinz C. Schröder, Werner E. G. Müller

**Affiliations:** 1 ERC Advanced Investigator Grant Research Group at the Institute for Physiological Chemistry, University Medical Center of the Johannes Gutenberg University, Duesbergweg 6, D-55128 Mainz, Germany; 2 Department of Dermatology, University Clinic Mainz, Langenbeckstr. 1, D-55131 Mainz, Germany; 3 Shenzhen Lando Biomaterials Co., Ltd., Building B3, Unit 2B-C, China Merchants Guangming Science Park, Guangming District, Shenzhen 518107, China; 4 Clinic for Oral and Maxillofacial Surgery and Plastic Surgery, University Medical Center of the Johannes Gutenberg University, Augustusplatz 2, D-55131 Mainz, Germany

**Keywords:** amorphous calcium carbonate, polyphosphate, wound healing, granulation tissue, metabolic energy, cell migration, microvascularization, diabetic mice, human epidermal keratinocytes

## Abstract

Amorphous calcium carbonate (ACC), precipitated in the presence of inorganic polyphosphate (polyP), has shown promise as a material for bone regeneration due to its morphogenetic and metabolic energy (ATP)-delivering properties. The latter activity of the polyP-stabilized ACC (“ACC∙PP”) particles is associated with the enzymatic degradation of polyP, resulting in the transformation of ACC into crystalline polymorphs. In a novel approach, stimulated by these results, it was examined whether “ACC∙PP” also promotes the healing of skin injuries, especially chronic wounds. In in vitro experiments, “ACC∙PP” significantly stimulated the migration of endothelial cells, both in tube formation and scratch assays (by 2- to 3-fold). Support came from ex vivo experiments showing increased cell outgrowth in human skin explants. The transformation of ACC into insoluble calcite was suppressed by protein/serum being present in wound fluid. The results were confirmed in vivo in studies on normal (C57BL/6) and diabetic (db/db) mice. Topical administration of “ACC∙PP” significantly accelerated the rate of re-epithelialization, particularly in delayed healing wounds in diabetic mice (day 7: 1.5-fold; and day 13: 1.9-fold), in parallel with increased formation/maturation of granulation tissue. The results suggest that administration of “ACC∙PP” opens a new strategy to improve ATP-dependent wound healing, particularly in chronic wounds.

## 1. Introduction

Wound healing is a complex tissue repair process enabled by the regenerative potential of skin tissue. Basically, this process is similar in wounds in the skin epithelium and the oral mucosa, but with different kinetics and gene expression patterns [1]. The programmed phases underlying cutaneous wound healing involve four steps: rapid hemostasis, inflammation, cell proliferation/differentiation and migration, and finally remodeling, which includes both functional re-epithelialization and synthesis, cross-linking, and processing of collagen to restore the strength of the healing tissue [1,2]. In the first phase, which begins immediately after wound setting, vasoconstriction occurs and a fibrin clot is formed. A characteristic process is the accumulation of blood platelets, followed by enhanced platelet aggregation. The second phase begins with the ingrowth of inflammatory cells and the sequential infiltration of neutrophils, macrophages, and lymphocytes. The macrophages reach their peak during the transition to the regenerative phase, in parallel with increased cell proliferation [3]. This phase is characterized by the increased proliferation of epithelial cells, leading to re-epithelialization of the wound area. Biochemically, this is a very intense anabolic phase during which the extracellular matrix (ECM), structural macromolecules, and hydrogel-forming molecules are formed. The organization of the ECM and the formation of the granulation tissue at the site of injury are supported by capillary growth, which initiates progression to the final remodeling phase.

These processes can be impaired by a variety of pathological factors such as cardiovascular and metabolic diseases, tumor diseases, infections, and increasing age, leading to the development of chronic wounds [4]. Chronic wounds such as diabetic foot ulcers, bedsores (pressure ulcers), and venous leg ulcers represent a significant burden not only for the patients but also for the economy [5]. The treatment of these wounds is a medical challenge, and novel materials are urgently needed to accelerate the healing of such wounds. A large number of wound healing materials have been developed in recent years, including (among the currently preferred materials) hydrogels [6], in particular hydrogels that exhibit stimuli-responsive and self-healing properties [7,8], as well as multifunctional stimuli-responsive nanoparticles [9].

A more recently developed strategy for wound treatment is based on the energy demand of this process. Energy supply is an essentially prerequisite for physiological wound healing [10,11]. ATP is required for the induction of angiogenesis, proliferation and differentiation of keratinocytes, cell migration, and the re-epithelialization process. It was reported that the initial migration and proliferation processes of endothelial cells in vitro are ATP-dependent [12]. Furthermore, ATP has been identified as a chemotactic signal for the endothelial cells along which they migrate and form ring-type patterns during initial vascularization [12]. The extracellular concentration of ATP is, however, low (~50 µM) [13], and it cannot be generated solely via intracellular processes, since the cell density in some tissues or tissue regions, e.g., in cartilage and dermis, often drops to 20% or even less [14]. Therefore, the question of where the ATP in the ECM comes from arose, in particular, the ATP that does not function as a ligand for purinergic receptors but for energy-dependent reactions, e.g., kinase reactions in the extracellular space. As mentioned above, platelets are among the first blood components to reach the defect site [15]. These cell fragments, and this has been overlooked until recently, are filled with large amounts of inorganic polyphosphate (polyP), which is enriched in their cell organelles along with Ca^2+^, Mg^2+^, and Zn^2+^ ions [16,17]. This molecule, polyP, released from the platelets, can act as an extracellular source for ATP, as shown in a number of studies (reviewed in [18]).

Physiologically, polyP is an unbranched oligomer or polymer with a chain length of up to 1000 phosphate units; usually, the chains are less than 100 units long [16]. The phosphate units are linked to one another via energy-rich anhydride bonds, which are also present in ATP. Importantly, after enzymatic hydrolysis of these bonds with alkaline phosphatase (ALP) [19], Gibbs free energy is released, which is converted into metabolically usable energy via the phosphorylation of AMP to ADP [20]. Inhibitor studies revealed that it is adenylate kinase (ADK) that subsequently converts ADP into ATP [12].

These results raised our interest in this metabolic energy-delivering inorganic molecule, polyP. PolyP has been described as essential for bone, tooth, and cartilage formation (for a review, see [14]). PolyP nanoparticles, similar to those present in platelets, have been fabricated using a biomimetic procedure [21]. These particles proved to be morphogenetically active in in vitro bone, tooth, and cartilage systems and in vivo in critical size bone defects (reviewed in [14]). Based on promising in vitro studies showing that exposure of cells to polyP particles causes a strong upregulation of the expression of the genes encoding for collagen, a main structural component of the skin, wound healing studies were performed on animals [22]. In these studies, a statistically very significant acceleration of the progress of wound healing after application of the calcium salt of polyP was demonstrated in both normal and diabetic mice showing delayed wound healing. First studies on patients with chronic, therapy-resistant wounds showed promising results [23,24].

Now, in the present study, a different approach to wound healing is presented, which, in addition to polyP, uses a second inorganic material, amorphous calcium carbonate (ACC), a material that has not previously been used to accelerate skin repair. The motivation to conduct this study came from the results of studies showing that ACC is an excellent matrix to enhance the proliferation, differentiation, and function of bone-forming cells [25,26]. Previous studies showed that the amorphous mineral, ACC, can be stabilized by polyP, i.e., that molecule that provides the metabolic energy required for these regeneration processes [25]. PolyP suppresses the conversion of ACC to crystalline CaCO_3_ almost completely at percentages of 10% [*w*/*w*] and higher with respect to CaCO_3_; lower amounts of polyP are ineffective [25]. The polyP-containing amorphous CaCO_3_ particles (“ACC∙PP”) precipitated in the presence of polyP are biodegradable, thereby releasing Ca^2+^ ions; they are most likely disintegrated time-dependently by the action of ALP. The “ACC∙PP” particles even cause upregulation of the expression of this enzyme, as shown in the human osteogenic sarcoma cell line SaOS-2 and mesenchymal stem cells [25], in addition to carbonic anhydrase IX (CA-IX), the enzyme forming ACC in in vitro cell culture systems [27]. The osteogenic potential of “ACC∙PP” has also been demonstrated in vivo in animal studies, which showed enhanced bone healing efficiency compared to conventional materials [26] in addition to high biocompatibility [25].

Based on these promising results, it was then asked whether polyP-stabilized ACC (“ACC∙PP”) is also a suitable material for promoting the healing of skin wounds, especially chronic wounds. The rationale behind conducting this study was twofold. Firstly, calcium ions, a component of “ACC∙PP”, have been shown to have a decisive role in wound healing. This applies in particular to the healing of chronic wounds such as venous or diabetic foot ulcers [28,29]. Calcium ions promote the proliferation, differentiation, and migration of keratinocytes [30,31,32], as well as the proliferation and collagen deposition of fibroblasts [33,34]. In addition, calcium is a stimulator of angiogenesis both in vitro and in vivo [35,36,37]. The acceleration of wound healing, especially in chronic wounds, has been demonstrated by the use of calcium alginate dressings [38,39] or dressings containing calcium-releasing nanoparticles [40].

Second, wound healing requires the presence of the therapeutic agent, in this case ACC, over longer periods of time. PolyP stabilizes ACC. It has been shown that only after degradation of polyP does the metastable ACC rapidly transform into its stable polymorph calcite, which shows no biological activity and releases calcium only very slowly [25]. However, it is expected that the polyP-degrading ALP becomes active soon after skin injury, in the initial phase of wound healing, after leakage of serum from ruptured blood vessels, leading to a shift in the acidic pH of the normal skin towards a more alkaline pH [41], providing optimal conditions for ALP activity [19]. Thereafter, in the inflammatory phase, an acidic pH develops that inhibits the growth of pathogenic bacteria that prefer a neutral or alkaline environment, and later, during the proliferative phase (granulation, angiogenesis, and re-epithelization), the pH shifts again towards the more alkaline range, leading to ALP-mediated degradation of polyP that stabilizes the ACC particles. In addition, polyP can undergo coacervate formation [42]. Therefore, it was also asked whether the polyP-lacking ACC, resulting after enzymatic removal of the polyP component, can be stabilized by protein present in wound fluid or serum. This could be shown here. This could be particularly important for chronic wounds that require prolonged treatment.

In this study, the effects of “ACC∙PP” particles on wound healing were examined in both in vitro and in vivo experiments. It is shown that the “ACC∙PP” particles significantly stimulate cell migration in the endothelial cell tube formation assay in vitro. The increased migration propensity of cells in the presence of “ACC∙PP” particles was confirmed in the wound healing scratch assay and by measuring the outgrowth of cells from human skin explants. Based on the in vitro results, in vivo experiments were performed both with normal (C57BL/6) and diabetic (db/db) mice. These studies revealed that topical administration of the “ACC∙PP” particles on experimental wounds strongly accelerates the kinetics of wound healing, particularly in diabetic animals. The histological examination of tissue sections from “ACC∙PP”-treated wounds showed a marked increase in the content and stage of maturity of granulation tissue in the regenerating wound. The results suggest that ACC has promising properties for potential application in wound healing.

## 2. Materials and Methods

### 2.1. Materials

Polyphosphate (polyP; sodium salt; food grade, purity ≥ 99%,) with an average chain length of 40 phosphate units was purchased from Chemische Fabrik Budenheim (Budenheim, Germany). Na_2_CO_3_ (#222321), CaCl_2_·2H_2_O (#223506), fetal bovine serum (FBS; #F7942), human epidermal keratinocytes (HEK; #102-05A), human epidermal keratinocyte complete culture medium (#SCMK001), EpiGR human epidermal keratinocyte complete culture media kit (#SCMK001), ECM gel (#E6909; ECM Gel from Engelbreth-Holm-Swarm murine sarcoma), MTT (thiazolyl blue tetrazolium bromide; #M2128), and 96-well plates (#CLS3595) were from Sigma-Aldrich (Taufkirchen, Germany). Cultrex basal membrane extract (BME; growth factor reduced) was from R&D systems (Abingdon; UK). Human umbilical vein endothelial cells (HUVEC) and bovine brain extract (#CC-4098) were from Lonza (Köln, Germany).

### 2.2. Preparation and Stabilization of Amorphous Calcium Carbonate Particles

The polyP-stabilized amorphous CaCO_3_ particles (“ACC·PP”) were prepared as previously described [25] by rapidly mixing an aqueous CaCl_2_·2H_2_O solution and an aqueous Na_2_CO_3_ solution in the presence of 10% [*w*/*w*] polyP (sodium salt) at room temperature and an equimolar concentration ratio between Ca^2+^ and CO_3_^2−^. Briefly, 20 mL of 0.1 M NaOH was supplemented with 0.1 g of polyP (sodium salt) and 1.05 g of Na_2_CO_3_ and then diluted with 30 mL of deionized water. This solution was added to 50 mL of water containing 1.47 g CaCl_2_·2H_2_O. The obtained particles were collected, washed with acetone, and dried at room temperature.

Non-stabilized ACC (without polyP) was prepared by addition of 50 mL of a solution containing 1.47 g CaCl_2_·2H_2_O in 0.9% NaCl to 50 mL of a solution containing 1.05 g of Na_2_CO_3_ in 0.9% NaCl. In a further set of experiments, 20 mL of 0.1 M NaOH was added to 30 mL of a Na_2_CO_3_ solution containing 1.05 g of Na_2_CO_3_ before mixing with the CaCl_2_ solution. After stirring for 1 h, the particles were collected by centrifugation, washed three times with distilled water, and freeze-dried for 24 h.

In order to study the effect of protein on the composition of the precipitated material (content of ACC, vaterite and/or calcite), the Na_2_CO_3_ or Na_2_CO_3_/NaOH solutions were supplemented with FBS as follows. FBS (1 mL or 10 mL) was added to 49 mL or 40 mL of the Na_2_CO_3_ or Na_2_CO_3_/NaOH solutions, after dissolution of the Na_2_CO_3_. The particles obtained after combining with the CaCl_2_ solution, stirring, centrifugation, washing, and lyophilization are termed “ACC-Ser-1” or “ACC-Ser-10” (1% [*w*/*w*] or 10% [*w*/*w*] FBS) and “ACC-Ser-1-NaOH” or “ACC-Ser-10-Na-OH” (1% [*w*/*w*] or 10% [*w*/*w*] FBS), respectively.

### 2.3. Fourier Transformed Infrared Spectroscopy

The Fourier transformed infrared spectroscopy (FTIR) was performed with ground powder in an ATR (attenuated total reflectance)-FTIR spectroscope/Varian 660-IR spectrometer (Agilent, Santa Clara; CA, USA), fitted with a Golden Gate ATR unit (Specac, Orpington, UK). The spectra were recorded in the range 500–4000 cm^−1^ with a resolution of 4 cm^−1^ (60 scans). The Varian 660-IR software package 5.2.0 (Agilent) was used for baseline correction, smoothing, and analysis, and Origin Pro (version 8.5.1; OriginLab, Northampton, MA, USA) for the graphical display and annotation of the spectra.

### 2.4. Cultivation of Epidermal Keratinocytes

Human epidermal keratinocytes (HEK) were cultivated in human epidermal keratinocyte complete culture medium as described [43]. The cultivation was carried out in 96-well plates at 37 °C. Before seeding, 200 µL of a suspension of the “ACC∙PP” particles in culture medium (50 µg/mL) or culture medium only (control) were added to each well. Then, the cells were added starting at 3 × 10^4^ cells per well, in a total volume of 0.8 mL.

### 2.5. MTT Cell Viability Assay

Keratinocytes were cultivated for 96 h and the cultures were then subjected to the cell viability MTT assay [44]. The cells were reacted with MTT and the color of the developed insoluble formazan product was read at 570 nm (Varioskan Flash; Thermo Fisher Scientific, Schwerte, Germany). Ten parallel, independent assays were performed.

### 2.6. HUVEC Cell Culture

Human umbilical vein endothelial cells (HUVEC; before passage 12) were cultivated in Endothelial Cell Basal Medium-Plus (Lonza) supplemented with 4% [*v*/*v*] FBS and 0.4% bovine brain extract (Lonza). As previously described, the cells were passaged every 3 days [45].

### 2.7. In Vitro Angiogenesis (Tube Formation) Assay

The assay was performed as described [46,47]. The plates (7 mm × 70 mm) were coated with Reduced Growth Factor Basement Membrane Extract (Trevigen, Gaithersburg: MD, USA) supplemented with 50 µg/mL of ACC∙PP. The coated plates were overlayed with HUVEC cells (density of 5 × 10^3^ cells per cm^2^). After 6 h and 12 h of incubation in Endothelial Cell Basal Medium, samples were processed through critical point fixation steps and inspected by ESEM (see below). The Wimasis Image Analysis software (Wimasis GmbH, München, Germany) was used to quantify the total tube area per field.

### 2.8. In Vitro Scratch Assay

The scratch assay was performed as previously described [48,49]. Petri dishes (60 mm; Nunc-Thermo Fisher Scientific, Dreieich, Germany) were coated with Cultrex basal membrane extract (BME) supplemented with 50 µg/mL of ACC∙PP by incubating the dishes overnight at 4 °C and overlaid with Endothelial Cell Basal Medium/FBS. After 10 min, the cells (HUVEC) were seeded onto the respective gel at a density of 8 × 10^3^ cells/cm^2^ and incubated further until the cells on the surface of the gels reached ~85% confluency. A scratch was made with a sterile 200 μL pipette tip. The cultures were then incubated for 8 h or 24 h and analyzed by light microscopy (phase contrast) (EVOS-XL, AMG, Thermo Fisher Scientific). Quantitative evaluation of the cell coverage was performed using the Image-Pro Plus software (Media Cybernetics, Rockville, MD, USA).

### 2.9. Cultivation of Skin Explants onto “ACC∙PP” ECM Gel

Ethical approval and consent was granted (§ 14 Abs.3 AVB of the University Medical Center Mainz). Skin explants with a diameter of 4 mm were removed from human dermis/epidermis samples with a diameter of 4 mm using a biopsy punch (pfm medical, Köln, Germany). The specimens were kept for a maximum of 6 h in EpiGR human epidermal keratinocyte complete culture media kit as described [50]. For the experiments, the explants were placed in an ECM gel warmed to a temperature of 5 °C. The gel was supplemented with 50 µg/mL of “ACC∙PP”, or left without “ACC∙PP” for the controls. A 1 mL aliquot was pipetted into each of the 24-well plates, and the explants (one specimen per well) were inserted into the gel and the assays were incubated for 24 h at 37 °C in the cell incubator. At 37 °C, the ECM gel formed a solid matrix. Microscopic examination was performed 6 h or 24 h after adding the explants to the gel.

### 2.10. Microscopic Analyses

Light microscopy was performed with a VHX-600 Digital Microscope (Keyence, Neu-Isenburg, Germany) equipped with a VH-Z100zoom lens. The scanning electron microscopic studies (SEM) were performed with HITACHI SU8000 electron microscope (Hitachi, Krefeld, Germany) and the ESEM (environmental scanning electron microscope) analyses with an ESEM XL-30 apparatus (Philips, Eindhoven, The Netherlands) as described [51].

### 2.11. Tissue Samples from the Mice Study with ACC∙PP

The details of the wound healing animal study (six animals per group [aged 6 and 7 weeks from Charles River, Calco; Italy]) with genetically diabetic male mice, BKS.Cg-m+Lepr^db^/+Lepr^db^ (db/db), and the common inbred strain of laboratory mouse, male C57BL/6, have been described previously [22]; ethical approval was granted; CAREZG_13-06-14_49 EP/2016 (SP-013-16). Wounds were set in the interscapular region, taking the precautions outlined [22]. After setting a single full-thickness excisional wound with a diameter of 8 mm, the “ACC∙PP” particles in powder form (3 mg; 100%) were administered directly into the wound beds immediately post-wounding (day 0). The controls received no additional wound medication. The wounds were covered by Tegaderm Wound dressing (3M, St. Paul, MN, USA), which remained in place until the end of the study.

After a healing period of 7 d or 13 d, the animals were sacrificed and biopsies of 1 cm × 2.5 cm (rectangular shape) were taken post mortem and transferred into 10% formalin for histological assessment. Slides were cut from paraffin blocks and stained with hematoxylin-eosin [52]. The morphometric evaluation of the degree of re-epithelialization and the amount of granulation tissue was performed using a Zeiss Axioskop 2 Plus microscope and applying an Axiovision program (Zeiss, Oberkochen, Germany) with a magnification of 100×. The re-epithelialization is expressed as the length of the newly formed epithelium (in mm) and the percentage of the wound diameter covered with a new epithelial layer [22]. The granulation tissue score is expressed as a percentage of the wound bed occupied by each of three granulation tissue categories (cellular—cellular, loose granulation tissue without collagen bundles; organized—tissue, in which fibroblasts and collagen bundles lie tight and parallel to each other while blood vessels run perpendicular from the bottom/edges towards the wound surface; and granulation tissue with collagen—tissue, where collagen predominates and cells are scarce). A mean value was calculated for the granulation tissue score for each experimental group.

### 2.12. Acute Skin Irritation

The study plan was reviewed by the Ethical Committee (CARE–Zagreb) in accordance with international laws/regulations and the Croatian Animal Protection Act (Official Gazette, NN 37/13) and the Animal Welfare Officer. The study was conducted in an AAALAC I-approved facility and the procedures were based on the OECD Guidelines for Testing of Chemicals, Section 4; Test No. 404: Acute Dermal Irritation/Corrosion, 24 April 2002; and International Standard: ISO 10993-10:2010, Biological evaluation of medical devices—Part 10: Tests for irritation and skin sensitization.

The study was performed using New Zealand white rabbits. Two animals were used. The fur was removed about 24 h before the test. The test substance (0.5 g “ACC∙PP” particles; as a powder) was applied directly to the test sites on the skin on each side of each rabbit. The application sites were covered with a 2.5 cm × 2.5 cm gauze patch, which was fixed with non-irritating adhesive tape for 5 h. At the end of the exposure period, the remaining test substance was removed with water. Untreated skin areas served as a control. The animals were euthanized on day 4.

All animals were examined for signs of erythema and edema. Dermal reactions were scored 60 min and then 24, 48, and 72 h after patch removal (No erythema/edema: irritation score 0; Very slight erythema/edema: 1; Well-defined erythema/edema: 2; Moderate erythema/edema: 3; Severe erythema/edema: 4).

### 2.13. Statistical Analysis

Student’s *t*-test and Mann–Whitney U test were used [53]. Values are expressed as the mean (standard error of the mean) or median.

## 3. Results

### 3.1. Preparation and Characterization of the “ACC∙PP” Particles

**Figure 1:**
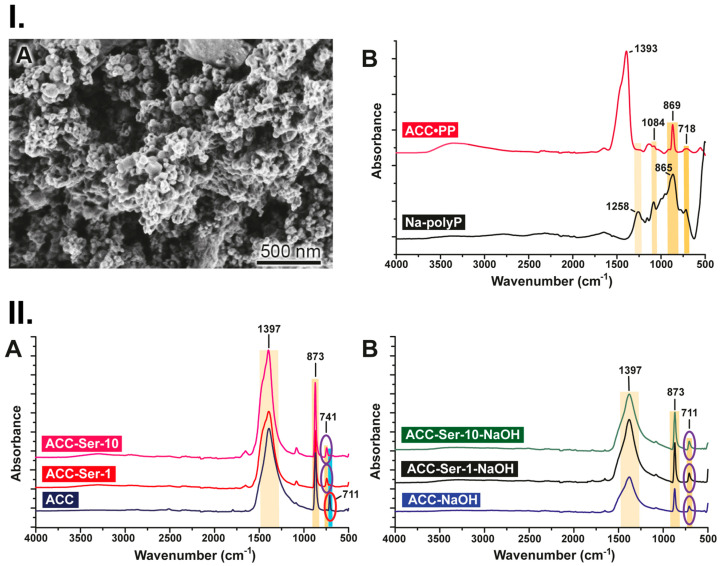
Polyphosphate-stabilized amorphous calcium carbonate (“ACC∙PP”) and effect of protein/serum. (**I**) Characterization of the “ACC∙PP” particles. (**A**) SEM. (**B**) FTIR spectrum of “ACC∙PP” in comparison to polyP (sodium salt; “Na-polyP”). The characteristic signatures for ACC and polyP are marked. (**II**) Suppression of transformation of metastable ACC into stable calcite at the vaterite stage. (**A**) FTIR spectra of “ACC” prepared without NaOH addition in the absence or presence of 1% [*w/w*] or 10% [*w/w*] serum (“ACC-Ser-1” and “ACC-Ser-10”). (**B**) FTIR spectra of “ACC-NaOH” prepared with NaOH supplementation in the absence or presence of 1% [*w*/*w*] or 10% [*w*/*w*] serum (“ACC-Ser-1-NaOH” and “ACC-Ser-10-NaOH”). The FTIR signal at 711 cm^−1^, indicative of calcite, and the signal for vaterite at 741 cm^−1^ are marked.

The “ACC∙PP” particles were prepared as described [25] by direct precipitation from aqueous solutions of CaCl_2_ and Na_2_CO_3_ in the presence of polyP. The sizes of the “ACC∙PP” particles varied around 500 nm (503 ± 137 nm) (Figure 1I(A)). The transition from the amorphous phase of CaCO_3_ to the crystalline phase is suppressed by adding 10% [*w*/*w*] polyP (0.1 g polyP [sodium salt] per 1 g of CaCO_3_) to the reaction mixture, as shown previously [25]. The FTIR spectrum of “ACC∙PP” in comparison to the spectrum of polyP (sodium salt) is shown in Figure 1I(B). In the spectrum of polyP (sodium salt), the characteristic polyP bands can be seen at 1258 cm^−1^ (ν_as_ for P=O), 1085 cm^−1^ (ν_as_; O-P-O), 917 cm^−1^ (ν_as_; P-O-P), and 864 cm^−1^ (ν_sym_; P-O-P). The spectrum of “ACC∙PP” shows peaks at 1398 cm^−1^, 869 cm^−1^, and 741 cm^−1^, which correspond to the absorption bands for vaterite at about 1080 cm^−1^ (ν_1_ symmetric stretching), 870 cm^−1^ (ν_2_ out of-plane bending), 1400 cm^−1^ (ν_3_ doubly degenerate planar asymmetric stretching), and 700 cm^−1^ (ν_4_ doubly degenerate planar bending). The absorption peaks in the range between 1200 cm^−1^ and 950 cm^−1^ of the “ACC∙PP” spectrum correspond to the phosphate bands of the polyP molecules.

### 3.2. Interaction of Serum with ACC Particles

As previously shown, polyP hydrolysis of the polyP component of “ACC∙PP” mediated by the ALP exopolyphosphatase activity [19] leads to the transformation of ACC into calcite, which is characterized by extremely low solubility [25]. Only CaCO_3_ particles formed in the presence of polyP (“ACC∙PP”), but not calcite, are biodegradable and disintegrated time-dependently both in vitro in cell culture and in vivo in rats, inducing gene expression and supporting cell/tissue regeneration [25]. Consequently, it can be expected that under these conditions, the administration of “ACC∙PP” to wounds does not result in the release of significant amounts of Ca^2+^ ions that support the wound repair process. In order to investigate whether under conditions found in the wound bed, in the presence of protein-containing wound fluid/serum, polyP-depleted ACC is stabilized by protein, the ACC precipitation was carried out in a reaction mixture containing serum (FBS) with or without NaOH supplementation. An increase in pH during precipitation has been shown to stabilize the ACC preparation [54,55]. ACC was prepared from CaCl_2_ and Na_2_CO_3_ at an equimolar ratio (0.1 M/0.1 M) of Ca^2+^ and CO_3_^2–^ in both the absence (“ACC”) and the presence of 0.02 M NaOH (“ACC-NaOH”). Both preparations were characterized by the presence of a significant amount of calcite (almost insoluble), but not vaterite, which has a low but significant solubility [25] (Figure 1II(A,B)). However, when FBS was added at a final concentration of 1% [*w*/*w*] or 10% [*w*/*w*] (“ACC-Ser-1” and “ACC-Ser-10”) during the ACC precipitation, the FTIR signal at 711 cm^−1^, which is indicative of calcite, disappeared and only the signal for vaterite at around 741 cm^−1^ was seen (Figure 1II(A)). This change was not observed when FBS was added during the preparation of ACC in the presence of NaOH (“ACC-Ser-1-NaOH” and “ACC-Ser-10-NaOH”); the calcite signal at 711 cm^−1^ remained and no signal at 741 cm^−1^ (vaterite) was visible (Figure 1II(B)). It is concluded that the suppression of calcite formation from the ACC particles at the vaterite stage by serum protein markedly increases the ability of the preparation to release Ca^2+^ ions, as previously demonstrated [25].

### 3.3. Effect on Growth/Viability of Epidermal Keratinocytes

The keratinocytes were seeded onto the wells of the culture plate and incubated in the absence or presence of “ACC∙PP” suspended in the culture medium (final concentration 50 µg/mL), as described in Materials and Methods. Quantitative analysis was performed using the MTT viability test. It was found that the cell density, as reflected by the color/formazan determination, only increased from 0.17 ± 0.04 (absorbance units) to 0.24 ± 0.04 after an incubation period of 2 d and to 0.52 ± 0.07 after 4 d in the cultures without “ACC∙PP”. When, however, the cells were grown in the presence of “ACC∙PP”, the viability level increased significantly (* *p* < 0.01) 2.1-fold (0.35 ± 0.09) or 1.5-fold (0.74 ± 0.07) after an incubation period of 2 d and 4 d, respectively.

### 3.4. Activation of HUVEC Cell Migration in the Scratch Assay

**Figure 2:**
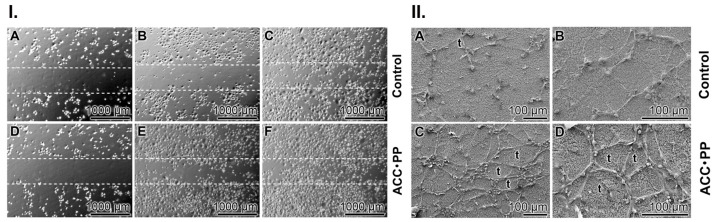
Effect of “ACC∙PP” on endothelial cell migration and tube formation. (**I**) Migration activity of HUVEC cells onto the ECM matrix supplemented (**A**–**C**) without or (**D**–**F**) with “ACC∙PP” in the in vitro scratch assay. The dashed lines mark the borders of the area scratched out from cells at the beginning of the experiments. The images (phase contrast light microscopy) were acquired after 0 h, 8 h and 24 h. It is obvious that only the scraped area in the assay with “ACC∙PP” was colonized again with the cells. (**II**) Formation of tube-like patterns by HUVEC on PEG-based hydrogels. HUVEC cells were plated onto (**A**,**B**) solubilized basement membrane extract without or (**C**,**D**) supplemented with “ACC∙PP” and incubated for 6 h or 12 h. The cell pattern was assessed by ESEM. Some complete tube-like structures (t) are marked.

The cell migration of HUVEC was determined in the scratch assay. The cells were incubated in a Petri dish coated with the basal membrane extract, which was supplemented either with “ACC∙PP” or remained without any addition of particles. After 8 h and 24 h, the migration of the cells into the scratched zone was first analyzed microscopically (Figure 2I). The number of cells that migrated into this region was strikingly different in the two assays. In both assays, only a few cells migrated into the scraped areas during the first 8 h (Figure 2I(A,B and D,E)). However, when the cultures were incubated for 24 h, only a few more cells migrated into the damaged zone in the assays without “ACC∙PP” (Figure 2I(C)). In contrast, the propensity to migrate was vigorous in the assays supplemented with the polyP-stabilized particles, “ACC∙PP” (Figure 2I(F)); the scraped area was almost completely re-colonized.

Using an image processing system, it was determined that after an incubation period of 8 h, the cells migrated into a covered area amounting to 51.0 ± 7.1% in the “ACC∙PP”-containing assays, while in the control assays, only about 18.1 ± 4.0% of the cells migrated into this area. After a prolonged incubation period of 24 h, an almost complete closure of the defect in the assay with “ACC∙PP” was seen.

### 3.5. Effect on Tube Formation of HUVEC Cells

Another very striking result was the difference of the potency of HUVEC cells for tube formation when seeded onto the growth factor basement membrane extract supplemented without or with “ACC∙PP” (Figure 2II). While in the “ACC∙PP”-lacking control, the propensity of the cells for tube formation was very low during the 6 h/12 h incubation period (Figure 2II(A,B)), the tube pattern forming activity by the HUVEC cells grown on the “ACC∙PP”-containing basement membrane extract was high (Figure 2II(C,D)). Especially high was the tube formation activity on the “ACC∙PP”-supplemented extract after 12 h of incubation (Figure 2II(D)).

The quantitative evaluation of the effect of the “ACC∙PP” particles showed that during an incubation period of 6 h (12 h), the number of tubes per visual field in the assays with cells growing on the “ACC∙PP”-supplemented basement membrane extract increased significantly to 27.3 ± 6.9 (38.9 ± 8.3) compared to the “ACC∙PP”-free controls with 8.7 ± 2.3 (17.2 ± 3.7).

### 3.6. Outgrowth of Cells from Skin Explants

**Figure 3:**
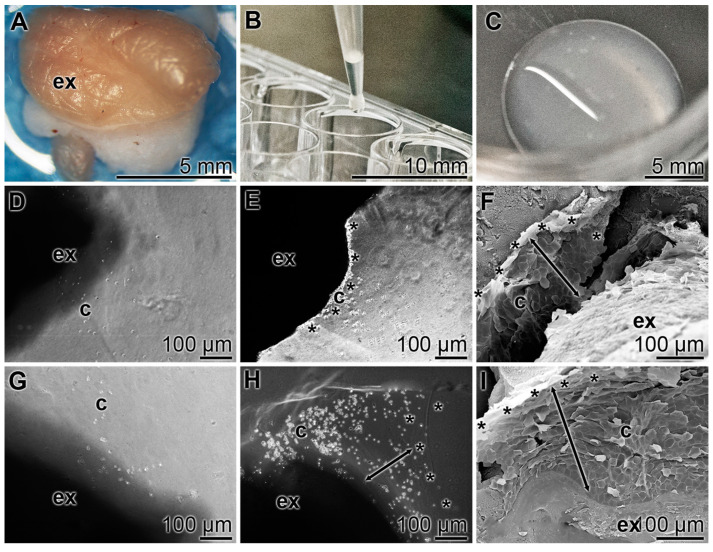
Outgrowth of cells from skin explants in the absence or presence of 50 µg/mL “ACC∙PP” particles. (**A**) Skin explants (diameter of 4 mm) were taken from human skin from the dermis/epidermis. (**B**) The explant samples were inserted into the ECM gel matrix pipetted into the 24-well plate. The gel was either without or enriched with “ACC∙PP” particles. (**C**) During the warming process to 37 °C, the gel became solid. (**D**–**F**) Outgrowth of cells from explants (ex) without “ACC∙PP” during an incubation period of 6 h or 24 h. The rims of the explants where cells (c) accumulate are marked (*) and their widths are highlighted with double-headed lines. (**G**–**I**) Outgrowth of cells from explants onto “ACC∙PP” particle-containing ECM gel. Light microscopy (**D**,**E**,**G**,**H**) and ESEM (**F**,**I**).

In order to assess the biological activity of cells in skin explants, these samples (diameter of 4 mm; Figure 3A) were taken; they contained both the epidermis and the dermis layer. The experiments were performed in the absence or presence of “ACC∙PP”. In the latter series, the ECM gel in the liquid phase (~5 °C) was supplemented with 50 µg “ACC∙PP” particles per mL of gel prior to insertion of the explants into the gel matrix. Then, 1 mL of gel was pipetted into each well of the 24-well plate (Figure 3B). Subsequently, the explants were inserted and the incubation continued at 37 °C. During warming, the gel solidified (Figure 3C). The cells migrating out of the explants during the 6 h and 24 h incubation periods of the explants on ECM gel were determined. In the absence of “ACC∙PP”, only a small rim with cells was seen at the borders of those explants (Figure 3D,E), while a considerably more expanded cell-containing rim was observed around the explants cultivated onto the gel containing “ACC∙PP” (Figure 3G,H). ESEM analysis revealed that the cells seen at the rim of the explants consisted mainly of squamous, keratinized cells characteristic of keratinocytes (Figure 3F,I).

### 3.7. Assessment of Wound Regeneration In Vivo

**Figure 4:**
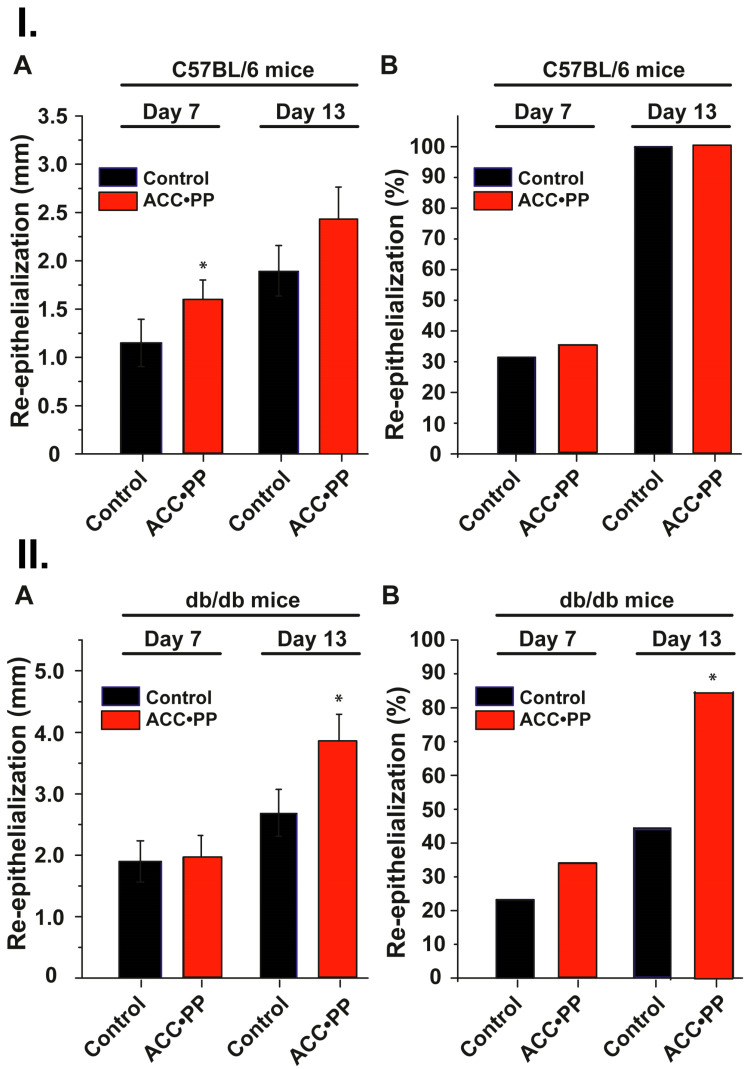
Effects of “ACC∙PP” particles on wound healing in C57BL/6 and db/db mice. (**I**) Normal C57BL/6 mice. (**A**) Degree of re-epithelialization (in mm); (**B**) Re-epithelialization (in percent). (**II**) Diabetic db/db mice. (**A**) Degree of re-epithelialization (in mm); (**B**) Re-epithelialization (in percent). A round wound of 8 mm was inflicted through the panniculus carnosus muscle in the interscapular region of the upper back of each mouse. After 7 d or 13 d, the wounds were inspected on the basis of re-epithelialization (in percent), which is calculated by dividing the degree of re-epithelialization (in mm) by the wound diameter (mm) × 100. In one series, the wounds did not receive any wound medication (controls); in the second “ACC∙PP” group, the wounds were treated with “ACC∙PP” micro-nanoparticles in the powder form. The data are presented as mean ± SEM (**A**) or median (**B**). The significance is * *p* < 0.05; n = 6 animals (Student’s *t*-test) [* *p* < 0.05 versus negative control according to the Mann–Whitney U test].

Wounds of 8 mm were set in the interscapular region of male mice, both normal C57BL/6 and db/db, as described in “Materials and Methods”. After a healing period of 7 d and 13 d, biopsies were taken from the wound/regeneration area and assessed morphometrically. During the 7-d healing period, the degree of re-epithelialization in the normal mice (C57BL/6) was 1.15 ± 0.24 mm (31.3%) in the controls and 1.60 ± 0.20 mm (35.2%) in the “ACC∙PP”-treated group (Figure 4I(A)). In the delayed wound healing model, the diabetic db/db mice studied in comparison to the normal mice, the degree of re-epithelialization on day 7 in the controls was only 1.89 ± 0.30 mm (22.9%), while a value of 1.97 ± 0.33 mm (34.3%) was determined in the “ACC∙PP”-treated animals (Figure 4II(A)). Much more pronounced were the changes in the degree of re-epithelialization in the diabetic animal group on day 13. While in normal mice, a complete or nearly complete re-epithelialization was seen in both the controls and the wounds treated with “ACC∙PP” (100%; Figure 4I(B)), the wounds in the nontreated group of db/db mice (controls) were only 44.8% re-epithelialized (Figure 4II(B)). However, the administration of “ACC∙PP” to the wounds of the diabetic mice resulted in significant acceleration in the healing of these (delayed healing) wounds when assessed by the rate of re-epithelialization. After a healing period of 13 days, these wounds were 84.4% covered by newly formed epithelium (Figure 4II(B)).

**Figure 5:**
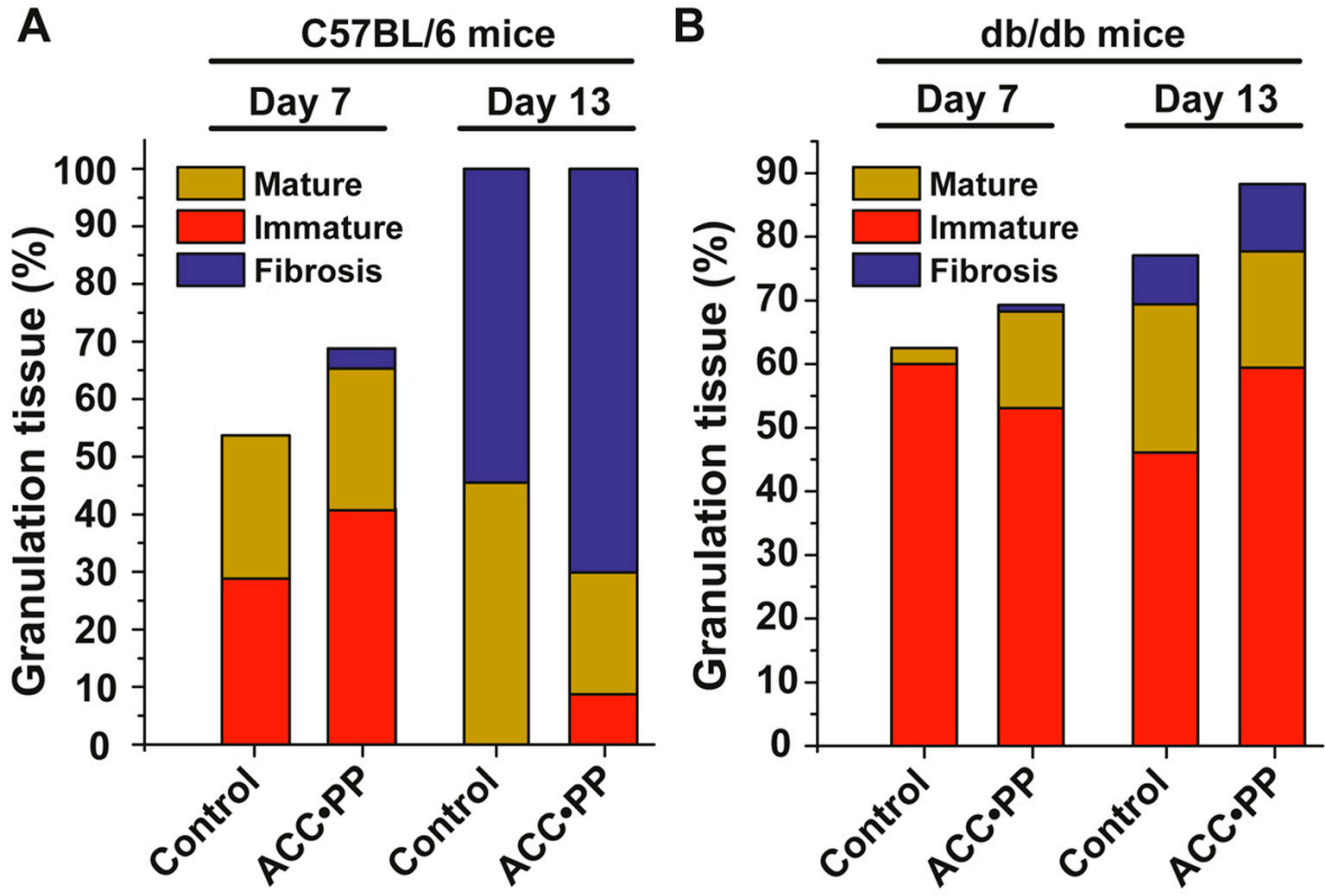
Effects of “ACC∙PP” particles on granulation tissue formation in C57BL/6 and db/db mice compared to non-treated controls. (**A**) C57BL/6 mice; (**B**) db/db mice. Shown are the percentage changes in total granulation tissue on day 7 and day 13, as well as the content of “immature” and “mature” granulation tissue (characterized by elongation of fibroblasts, early deposition of collagen fibers, and angiogenesis) and fibrosis.

Analysis of the granulation tissue in the wounds of non-treated diabetic control mice on day 7 revealed a higher content of immature tissue compared to wild-type C57Bl6 mice (Figure 5A,B). The treatment of the mice with “ACC∙PP” resulted in a marked increase in the content of mature granulation tissue in expense of the immature tissue in the diabetic animals (Figure 5B). The changes in wild-type mice were less pronounced (Figure 5A). On day 13, the wound surface in wild-type mice was totally (100%) covered with granulation tissue, both in untreated and “ACC∙PP”-treated mice, with a significant increase in the content of fibrosis and a decrease in mature tissue (Figure 5A), while in db/db mice, the “ACC∙PP” exposure led to an increased formation of granulation tissue from about 80 to 90% of the wound surface, along with an enhancement in fibrosis (Figure 5B).

### 3.8. Acute Dermal Irritation

The administered dose was 0.5 g test substance (“ACC∙PP”) per test site. The study (control and “ACC∙PP”) on two New Zealand white rabbits showed no clinical signs of erythema and edema at 1 h, 24 h, 48 h, and 72 h. No eschar formation was observed. All gratings were 0/0. The differences in body weight observed on day 1 and day 4 were consistent with the initial weights. Based on the result obtained, “ACC∙PP” is not considered a primary skin irritant.

## 4. Discussion

Intensive efforts are currently undertaken in order to bring the duration and severity of wound healing, particularly in patients with local/systemic diseases, to a physiological level by promoting the regenerative processes. As described in the Introduction, it is known that the energy status in regenerating wound tissue correlates with the kinetics of wound healing. ATP is the most important metabolite in wound regeneration both intra- and extracellularly. It is known that the release of ATP/ADP from stimulated blood plates increases after tissue damage [13] and that ATP from the glycolytic pathway contributes to microvascularization [56] and chemotactic migration of endothelial cells [12]. The most convincing results are those that show that both the direct administration of ATP [57] and the extracellular administration of polyP, followed by an increased ADP/ATP level, significantly accelerate wound healing in vivo [22].

The present study describes the biochemical basis and mode of action of “ACC∙PP” as a novel, bioactive physiological inorganic material, which speeds up wound healing based on its properties that activate the migration of cells both from skin explants and artificial scratch/wound healing assays and initiate microvascularization. The “ACC∙PP” particles were prepared by precipitating CaCO_3_ in the presence of polyP using our previous protocol [25]. The particles are spherical and amorphous, as already shown [25]. The integration of the polyP component in these particles is visible in the FTIR spectra, which show the characteristic signatures for polyP in addition to the ACC signals.

Based on the presented results showing that “ACC∙PP” enhances energy-dependent regeneration/repair processes and supporting the view that polyP positively influences the energy status of cells and tissues [20], it is concluded that the polyP component of the “ACC∙PP” particles contributes significantly to the beneficial effects of these particles during wound healing.

The main component of these particles, ACC, is a material that is physiologically formed by the enzyme carbonic anhydrase (CA) [58], more precisely the cell membrane-associated CA IX [14]. Interestingly, the expression of the CA IV and CA IX genes increases during wound healing, especially during the initial inflammatory phase due to vascular disruption and impaired oxygen delivery [59]. Previously, it has been shown that ACC and bicarbonate (HCO_3_^–^), which is liberated during the disintegration/dissolution of ACC, induce the expression of CA in bone-forming SaOS-2 cells [27]. Using SaOS-2 cells and MSC, it was found that “ACC∙PP” also enhances the expression of ALP [25], an effect at least partially caused by the presence of the polyP [26].

The metastable ACC undergoes rapid transformation into the crystalline polymorphs vaterite, calcite, or aragonite [60]. The type of polymorph formed depends on a number of factors such as temperature, metal ions, and organic molecules [61]. At lower temperatures, below 30 °C, ACC mostly transforms into the calcite stage via the intermediate formation of vaterite, while at higher temperatures, above 60 °C, aragonite is formed, again via the vaterite stage [62]. In human bone formation, ACC can also be converted to amorphous calcium phosphate, depending on the availability of exogenous phosphate [27]. Based on model simulations, it was recently proposed that ACC consists of a hierarchically organized gel-like structure of anhydrous CaCO_3_ nanodomains embedded in a matrix of network-like arranged water molecules [63]. This could explain the stabilization of ACC by polyelectrolytes, both protein and polyP as shown here, which could enter the aqueous channels of this structure.

In nature, platelets are the dominant source of polyP [64]. The detailed chemical structure, including linear/cyclic molecules, and counterions of the polyP enriched in these cell organelles, is not yet known and needs further studies (for a discussion of available methods and their limitations, see [65]). Platelet polyP is released upon activation at damaged tissue sites, including skin defects [23]. The stabilization of ACC, similar to polyP, has also been observed for extracts from gastroliths containing phosphate-rich organic matrix proteins [66]. Using the formation of sponge spicules as a model, it has been shown that the formation of CaCO_3_ crystals can be blocked at the vaterite stage by an aspartate/glutamate-rich peptide (D/E peptide) present in spicules of the siliceous sponge *Suberites domuncula* [27].

In the study presented here, ACC was stabilized by polyP to prevent its rapid transformation into calcite and to allow calcium ion release over longer periods of time. ACC has a much higher solubility than calcite (by two orders of magnitude on the logarithmic scale) and can, therefore, serve as a source of Ca^2+^ ions to improve wound healing. The temperature of the skin surface (usually in the range 33.5–36.9 °C) can vary between 15 and 45 °C depending on the body region and after exposure to cold or hot environments [67]. The solubility product of ACC in this range is between 4.0 × 10^−7^ (25 °C) and 2.5 × 10^−7^ (40 °C) [68], while the solubility product of calcite (nearly insoluble) is between 3.3 × 10^−9^ (25 °C) and 2.63 × 10^−9^ (40 °C), calculated from the respective p*K* values; p*K* = 8.480 (25 °C) and p*K* = 8.580 (40° C) [69]. Vaterite, present in “ACC∙PP” in significant and increasing amounts over time (due to the blocking of ACC conversion to calcite at the vaterite stage), is slightly more soluble than calcite, with a solubility between 1.2 × 10^−8^ (25 °C) and 8.9 × 10^−9^ (40 °C) [p*K* = 7.913 (25 °C) and p*K* = 8.051 (40 °C)] [69]. To rule out that the polyP-free ACC, obtained after hydrolysis of the polyP component of “ACC∙PP” by the ALP present in wound fluid [24], is immediately transformed into almost insoluble calcite, which is not suitable as a source for the release of calcium ions, the effect of protein/serum on ACC was examined. The results showed that in the presence of protein/serum, the formation of calcite was suppressed (disappearance of FTIR signal at 711 cm^−1^) and only the signal for vaterite (at around 741 cm^−1^) was observed.

In the skin explant/migration experiments performed in the present study, an ECM gel prepared from Engelbreth-Holm-Swarm mouse sarcoma [70] was used as hydrogel matrix. This hydrogel, containing fibronectin, laminin, and collagen type I, mimics the ECM and inherently supports adhesion of cells to fibrils and cell differentiation and stimulates tubule formation due to the presence of intrinsic growth factors and transforming growth factors [71]. This gel was chosen to provide the cells with a largely physiological growth environment. The results showed that attaching the skin explant to the ECM gel supplemented with “ACC∙PP” markedly accelerated the outgrowth of cells from this tissue.

The stimulating activity of the “ACC∙PP” particles on energy/ATP-dependent cell migration could also be demonstrated in the endothelial cell tube formation assay with HUVEC cells and in the scratch assay as another in vitro wound healing model system. In both assays, a clear difference in cell migration propensity was observed when cells were exposed to the matrix containing “ACC∙PP” particles compared to cells without the particles. The increase in number of tubes per visual field in the tube formation assay with cells growing on the “ACC∙PP”-supplemented basement membrane extract amounted to 3.1-fold after an incubation period of 6 h (or 2.3-fold after 12 h) compared to the “ACC∙PP”-free controls. In the cell migration assay, an about 2.8-fold increase in the migration activity was observed during an incubation of 8 h.

In addition to the ACC component, which provides calcium ions, the polyP component of the “ACC∙PP” particles certainly contributes to the wound healing/regenerative activity of the particles. The effect of polyP on wound healing has also previously been demonstrated in in vivo animal studies [22]. PolyP functions as an extracellular metabolic energy source, a source of ATP formed by the combined action of ALP and ADK, as shown in inhibitor experiments using cell cultures [12,18,20]. It should be noted that ADP, the immediate product of the ALP phosphotransferase reaction, may also favorably affect wound healing. The activation of the P2Y_12_ receptor by binding ADP has been reported to accelerate wound healing in diabetic mice [72]. In addition to polyP, ADP is also released from platelets after activation. This nucleotide also increases collagen synthesis as well as proliferation/migration of fibroblasts and keratinocytes and differentiation of myofibroblast in the wound area [72]. It has also been reported that shock wave treatment can accelerate wound healing by inducing the release of cellular ATP that binds to purinergic receptors, leading to an enhanced cell proliferation through the activation of the Erk1/2 and p38 MAPK signaling pathways [73].

Two animal models were used to assess the effects of the “ACC∙PP” particles on wound healing in vivo, wild-type (C57BL/6) mice to assess the effects of the material on the rate of acute wound healing and diabetic (db/db) mice to study their—even more important—influence on the healing process of chronic wounds. In both comparative studies, the “ACC∙PP” particles were administered topically as a solid powder material. The db/db mice have a homozygous mutation of the leptin gene, which is involved in regulating energy balance, leading to hyperphagia, obesity, and diabetes [74,75]. It has been shown that this exact gene is induced by polyP in mouse calvaria MC3T3-E1 cells [76].

After a healing period of 7 days in the absence of “ACC∙PP”, re-epithelialization of acute wounds was about 31.5% complete in normal mice and 100% after 13 days, in contrast to diabetic mice, which showed delayed healing kinetics with 23% epithelialization after a period of 7 days and only 44.5% at the end of the study (day 13). This wound healing rate was significantly accelerated by the topical treatment with the “ACC∙PP” particles, especially in diabetic mice, as shown in the present animal study. While the stimulating effect of the test material on the re-epithelialization rate in diabetic mice was rather low (34%) after the 7-day healing period in the presence of “ACC∙PP”, the healing process was markedly accelerated to about 85% in the second half of the healing period and reached almost the same level as in the animals with normal wound healing.

The histological examination of the areas of the wounds untreated and treated with “ACC∙PP” particles showed a drastic increase in the epidermal layer and even more in the dermis layer of the wounds treated with “ACC∙PP” compared to controls, as well as an enhanced neovascularization, indicated by the appearance of an increased number of new blood vessels compared to the control. Overall, the relative increase in granulation tissue in mice treated with “ACC∙PP” compared to untreated control mice was significantly more pronounced in the diabetic db/db animal group than in the wild-type animal group. It should be noted that ALP, the enzyme that provides the metabolic energy needed for ECM synthesis and cell migration/vascularization in tissue regeneration [18], is highly active in the granulation phase [77] and can, therefore, mediate at least part of the beneficial effects of “ACC∙PP” during wound repair. This enzyme is also present in the wound fluid and reaches an activity 24–146 U/L (median: 84 U/L), similar to the enzyme in serum with 58–247 U/L (median: 120 U/L) [78].

The results presented here demonstrate for the first time that “ACC∙PP”, a material previously found to be regeneratively active in healing bone defects [25,26], is also effective in wound healing, both in vitro and in vivo. A similar strategy in wound treatment has not been addressed before (for a review of current materials, see [4]). Other strategies, e.g., based on the use calcium-releasing nanoparticles such as CaP ormoglass (organic modified calcium phosphate glass; [37]) also showed an accelerating effect on chronic wound healing [40], but these strategies lack the energy-providing effect of polyP. In principle, polyP alone in the form of Ca-polyP nanoparticles has also a stimulatory effect on wound healing, as shown previously [22]; however, in this case, after the ALP-mediated degradation of this molecule, no calcium-releasing store remains that can maintain the wound-healing effect of calcium ions. In contrast to the effect of “ACC∙PP” on bone regeneration, which certainly also includes the provision of starting materials for hydroxyapatite and carbonated hydroxyapatite formation (P_i_, Ca^2+^ and HCO_3_^−^ ions) [25,26], the effect on skin regeneration during wound healing is most likely based only on the Ca^2+^ ions acting as regulators promoting the healing process [28,29,30,31,32,33,34,35,36] and the supply of metabolic energy (ATP) by the polyP component of the particles.

**Figure 6:**
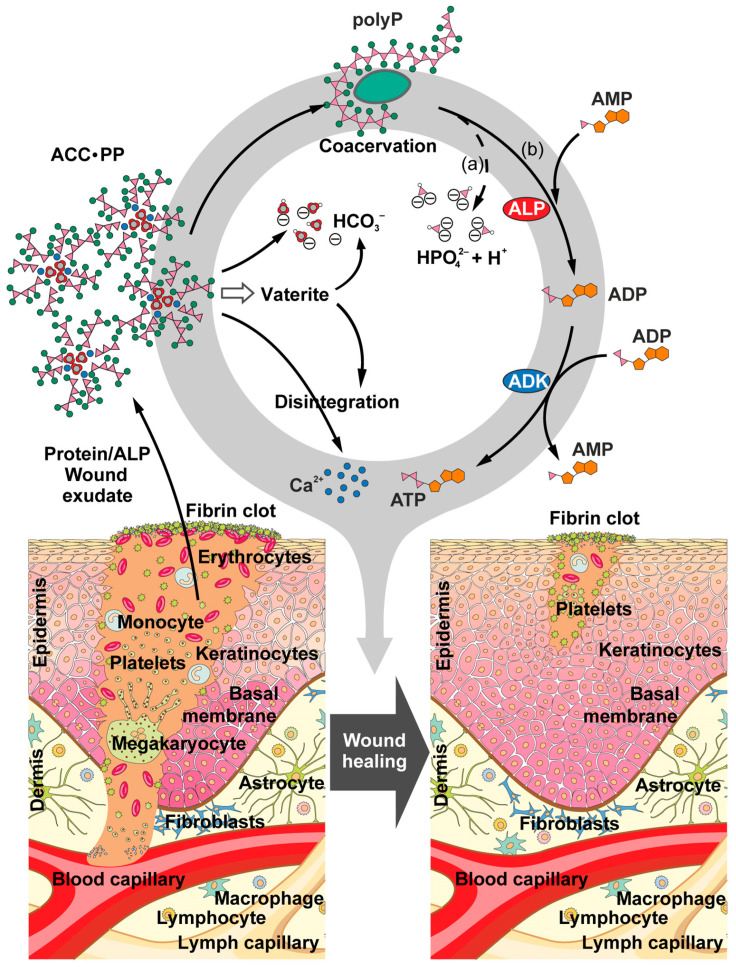
Sketch showing the proposed mode of action of “ACC∙PP” particles. Upon contact with wound exudate containing protein/alkaline phosphatase (ALP), the “ACC∙PP” particles disintegrate. The released polyP undergoes coacervation through interaction with positively charged protein segments, as well as ALP-catalyzed degradation to monomeric phosphate or phosphotransfer to AMP to form ADP, which is converted to AMP and ATP by cell membrane-associated adenylate kinase (ADK). The polyP-depleted ACC can be further stabilized by protein or decomposes to Ca^2+^ and HCO_3_^−^ (in acidic environments), which allows for pH balancing (a) and counteract acidification by ALP-mediated polyP hydrolysis (HPO_4_^2−^), creating optimal conditions for ALP activity. The generation of ATP (b) and the release of Ca^2+^ ions leads to an acceleration of the wound healing process, particularly important in chronic wound healing.

It is suggested that the mechanism behind the acceleration of wound healing by the “ACC∙PP” particles is as follows (Figure 6). These particles disintegrate after contact with protein-containing wound exudate, which also contains the polyP-degrading ALP [75]. PolyP can also undergo coacervate formation with cationic domains of the protein polypeptide chains [42]. The liberated ACC freed from the polyP component can be further stabilized by the wound fluid protein at the vaterite stage, thereby prolonging the period of releasing Ca^2+^ ions from the material over the healing period. In addition to polyP hydrolysis to monomeric phosphate, ALP is able to catalyze the formation of ATP via phosphotransfer to AMP and interconversion of the resulting ADP to AMP and ATP in a combined enzymatic reaction with ADK. The release of CO_3_^2−^/HCO_3_^−^ ions in addition to Ca^2+^ ions from ACC, which has a higher solubility than crystalline CaCO_3_ [79], especially in acidic environments, could help balance the pH, which may drop due to polyP hydrolysis, creating the neutral to slightly basic conditions required for optimal ALP activity. The generated ATP and the released Ca^2+^ ions then develop their promoting effects to support wound healing.

## 5. Conclusions

In conclusion, amorphous calcium carbonate particles stabilized by polyP (“ACC∙PP”) were prepared and their effect on wound healing was investigated both in vitro and in vivo. Experiments with an in vitro scratch assay and tube formation assay showed that “ACC∙PP” has the property of increasing the migration propensity of endothelial cells by 2–3-fold. These results were supported by ex vivo studies measuring the outgrowth of keratinocytes in human skin explants. PolyP is susceptible to degradation by ALP. It is shown that in the absence of polyP, the rapid conversion of the amorphous phase of calcium carbonate, ACC, to insoluble calcite is suppressed by protein/serum, suggesting that enzymatic degradation of polyP does not immediately lead to the formation of the most insoluble calcium carbonate polymorph that no longer functions as a suitable calcium source for wound healing. The results of the animal study show that “ACC∙PP” particles also efficiently accelerated wound healing in vivo, especially in diabetic mice. In these animals, a 1.5-fold (day 7) to 1.9-fold (day 13) increase in the rate of re-epithelialization was observed, parallel to an increased formation of granulation tissue. We conclude that the calcium ion- and energy-supplying as well as pH stabilizing/ALP activity-supporting “ACC∙PP” particles have the potential to become a promising material in topical wound therapy, especially in chronic wounds. These particles could be administered either directly or after supplementation in a gel or paste, or as part of a regenerative wound dressing.

## Data Availability

Data will be provided upon reasonable request.
